# Overexpression of TRIM24 Stimulates Proliferation and Glucose Metabolism of Head and Neck Squamous Cell Carcinoma

**DOI:** 10.1155/2018/6142843

**Published:** 2018-05-10

**Authors:** Hongming Wang, Weishuang Xue, Xuejun Jiang

**Affiliations:** ^1^Department of Otolaryngology, The First Affiliated Hospital of China Medical University, Shenyang, Liaoning, China; ^2^Department of Neurology, The First Affiliated Hospital of China Medical University, Shenyang, Liaoning, China

## Abstract

TRIM24 (Tripartite Motif Containing 24) is a recently identified oncogene overexpressed in various cancers. However, the molecular mechanism of TRIM24 in the progression of head and neck squamous cell carcinoma (HNSCC) remains ambiguous. In the present study, we analyzed the expression pattern of TRIM24 in 100 HNSCC tissues and found that TRIM24 was overexpressed in 43/100 HNSCC cases. Significant association was found between TRIM24 overexpression and tumor-node-metastasis (TNM) stage (*p* = 0.0034) and T stage (*p* = 0.0048). Furthermore, we overexpressed and knocked down TRIM24 in Detroit 562 and FaDu cell lines, respectively. TRIM24 overexpression promoted proliferation, colony formation, and invasion, while TRIM24 depletion inhibited proliferation, colony formation, and invasion. Further studies showed that TRIM24 facilitated cell cycle transition and upregulated cyclin D1 and p-Rb. In addition, we found that GLUT3, a key protein involved in regulating glucose metabolism, was altered in HNSCC cells overexpressing TRIM24. We demonstrated that TRIM24 overexpression increased glucose uptake ATP production. Overexpression of TRIM24 increases cell sensitivity to glucose deprivation in Detroit cells. Depleting TRIM24 in FaDu cells demonstrated the opposite results. We also showed that TRIM24 could bind to the promoter region of cyclin D1. In conclusion, TRIM24 is upregulated in HNSCC and promotes HNSCC cell growth and invasion through modulation of cell cycle, glucose metabolism, and GLUT3, making TRIM24 a potential oncoprotein in HNSCC.

## 1. Introduction

Laryngeal carcinoma is a common head and neck cancer, with more than 150 thousand new cases recorded and 82 thousand deaths estimated in 2008 [1]. During the last decade, treatments for laryngeal carcinoma including chemotherapy and radiotherapy greatly improved patient survival. However, chemotherapy and radiotherapy cause acute and chronic toxicities [2]. Therefore, a global genomic perspective is important to elucidate the underlying molecular mechanisms and characteristics of laryngeal carcinoma in order to further improve survival rates and treatments.

TRIM24 is composed of a TRIM (Tripartite Motif Containing 24) motif, a NR (Nuclear receptor) box motif, and a C-terminal region with PHD (Plant homeodomain) finger domain [3, 4], which is reported to regulate chromatin remodeling [5]. TRIM24 is able to regulate transcription factors in a ligand dependent manner. TRIM24 is reported to interact with RAR*α* (retinoic acid receptor, alpha) [3]. It also interacts with the activation function 2 (AF2) region of several nuclear receptors, including the estrogen, retinoic acid, and vitamin D3 receptors [4, 6, 7].

Recently, growing evidence implicated the involvement of TRIM24 in tumor progression. It is reported that TRIM24 is involved in oncoprotein fusion by chromosome translocation in various cancers including leukemia, thyroid carcinoma, and myeloproliferative syndrome [6, 8]. It is overexpressed in human breast cancer and correlated with poor patient prognosis [9, 10], indicating a potentially oncogenic function for TRIM24 in human cancers. There is one report showing TRIM24 is overexpressed in HNSCC and correlated with poor survival and apoptosis [11]. However, how TRIM24 regulates HNSCC cell proliferation, especially its effect on metabolism, still remains obscure. In order to address these questions, we examined TRIM24 expression in HNSCC tissues by immunohistochemistry. In addition, we also investigated the effect of TRIM24 on proliferation and invasion of HNSCC cells and explored possible mechanisms.

## 2. Materials and Methods

### 2.1. Specimens

Protocol of the present study was approved by the Institutional Reviewer Board of China Medical University. This study was conducted in accordance with the Declaration of Helsinki. 100 primary head and neck squamous cell carcinoma specimens were obtained from pathology archive of the First Affiliated Hospital of China Medical University between 2010 and 2014. Informed consent was obtained from all patients.

### 2.2. Immunohistochemistry

Tumor specimens were fixed with 10% neutral formalin, and 4 *μ*m thick paraffin sections were made. Immunostaining was performed using the S-P staining kit from MaiXin (Ultrasensitive™, MaiXin, Fuzhou, China). After antigen retrieval in citrate buffer (pH 6.0) for 2 min in an autoclave, 0.3% hydrogen peroxide was used for 15 minutes and then the sections were incubated with goat serum. TRIM24 rabbit polyclonal antibody (1 : 1000 dilution; Proteintech, USA) was incubated at 4°C overnight. Biotinylated goat anti-rabbit serum IgG was incubated after washing in PBS. Then streptavidin–biotin conjugated with horseradish peroxidase was incubated. DAB kit (MaiXin, Fuzhou, China) was used for staining.

TRIM24 staining was scored according to previous reports [12, 13]. Nuclear staining in tumor cells was considered TRIM24 positive staining. The intensity was divided as follows: 0, negative; 1, moderate; 2, strong. The percentage score was divided as follows: 0: 0%; 1: 1–25%; 2: 26–50%; 3: 51–75%; and 4: 76–100%. The two scores were multiplied to get a final staining score from 0 to 8. Tumor samples with score 4–8 were considered as TRIM24 overexpression.

### 2.3. Cell Culture and Transfection

FaDu and Detroit 562 cell line were purchased from Shanghai cell bank (Shanghai, China). STR testing result was also obtained from Shanghai cell bank, Chinese Academy of Sciences (Supplementary Figure 2). Cells were cultured using RPMI1640 (Invitrogen, Carlsbad, CA, USA) containing 10% FBS at 37°C in 5% CO_2_. The plasmid of TRIM24 was purchased from OriGene. For transient knockdown experiments, small interfering RNA (siRNA) targeting human TRIM24 (GAGCAUAGAUACCAAUUUA) and nontargeting siRNA were purchased from Dharmacon (ThermoFisher, Lafayette, CO, USA) and transfected using Lipofectamine 3000 (Invitrogen). The cells were transfected according to the manufacturers' protocol and were harvested 48 hours later.

### 2.4. Western Blot Analysis

Proteins were extracted from transfected cells using cell lysis buffer. Then protein quantity was examined by Bradford method. 50 *μ*g proteins were added to SDS-PAGE and then transferred to PVDF membrane from Millipore. Membranes with transferred proteins were incubated at 4°C for 12 hours with primary antibodies including TRIM24 (1 : 1000; Proteintech, USA), GLUT1 (Abcam, 1 : 1000), GLUT2 (Abcam, 1 : 600), GLUT3 (Abcam, 1 : 800), GLUT4 (Abcam, 1 : 1000), cyclin D1 (1 : 1000), p-Rb (1 : 1000), Rb (1 : 1000), and GAPDH (1 : 2000; Cell Signaling Technology, USA). After incubation with peroxidase-coupled anti-rabbit/mouse IgG (1 : 2000; Cell Signaling Technology, USA) at room temperature for 3 hours, ECL was used on membranes and protein bands were detected using Imaging System.

### 2.5. Real-Time PCR

RNA extraction was performed using RNAiso (TAKARA, China). Real-time PCR was performed using SYBR Green MasterMix from ABI with ABI 7500 system (Applied Biosystems, USA). A dissociation procedure was performed to generate a melting curve for confirmation of amplification specificity. *β*-Actin was used as the reference gene. Relative quantification of target genes was calculated using the 2^−ΔΔCt^ method.

### 2.6. Cell Counting Kit-8 (CCK-8)

Cells were plated in 96-well plates in MEM containing 10% serum at approximately 1000–1500 cells per well. For cell viability measurement, cultures were added with CCK8 solution. About 20 *μ*l of CCK-8 solution was added to each well and incubated for another 4 hours. Each well of 96-well plates was detected at 490 nm.

### 2.7. Colony Formation Assays

Cells were transfected with TRIM24 plasmid or siRNA. Cells were seeded into 6-cm dishes (about 2000 per dish). Then dishes were cultured for another 12 days. Dishes were washed and Giemsa was performed to stain and visualize colonies. Using microscope, we counted colonies with cells more than 50 cells.

### 2.8. Matrigel Invasion Assay

Cell invasion was examined using transwell assay with 24-well transwell chambers. Briefly, inserts of transwell chamber were coated using 20–25 *μ*l Matrigel from BD with a dilution rate of 1 : 5. About 48 hours after cell transfection, about 1 × 10^5^ cells resuspended in 100 *μ*l of serum-free medium were added to upper chamber. MEM with 10–15% serum was added to lower chamber. After 16–20 hours' incubation, cells on the upper side of membranes were removed and the cells that invaded the filter were washed with PBS and visualized with hematoxylin.

### 2.9. Glucose Deprivation

Cells were cultured in bottles and then 1 day later switched to glucose-free medium with 10% dialyzed FBS.

### 2.10. Cell Cycle Analysis

48 hours after transfection, cells were harvested, fixed in 1% paraformaldehyde, washed with PBS, and stained with 5 mg/ml propidium iodide in PBS supplemented with RNase A (Roche, Indianapolis, IN) for 30 minutes at room temperature. Cells in each individual phase of the cell cycle were determined based on their DNA ploidy profile using ACEA Flow Cytometer and NovoExpress software.

### 2.11. ATP Production and Glucose Consumption

ATP production was measured by ATP assay kit (Abcam). Glucose levels were determined using glucose assay kit (Biovision). Assays were performed according to the manufactures' instruction.

### 2.12. Chromatin Immunoprecipitation (ChIP)

ChIP was performed using Magna ChIP A/G kit (Millipore) according to the manufacturer's instructions. Briefly, 1 × 10^7^ cells were harvested, cross-linked with 1% formaldehyde on ice for 10 min, quenched with glycine for 5 min, and washed twice with cold PBS. Then the cells were lysed and sonicated to a manageable size. Lysates were then incubated with affinity-purified antibody (rabbit anti-TRIM24 antibody or rabbit IgG) and fully resuspended protein A/G magnetic beads overnight at 4°C with rotation. After elution of protein/DNA complexes and reverse cross-links of protein/DNA complexes to free DNA, DNA was purified and real-time PCR was performed. Data were presented as fold enrichment of the TRIM24 antibody signal versus the negative control IgG, calculated using the comparative Ct method. Specific primers for the TRIM24 site in the cyclin D1 promoter region are 5′-CACACCGAAGCCTCAGTTGC-3′ and 5′-CCCGCCTTTCTCTTCATCCCC-3′.

### 2.13. Statistical Analysis

SPSS 16 was used for all statistical analyses. *χ*^2^ test was performed to explore the correlation of TRIM24 overexpression with clinical parameters. *t*-test was used to compare other data. *p* < 0.05 was considered as statistically significant.

## 3. Results

### 3.1. TRIM24 Is Overexpressed in HNSCC Tissues

**Figure 1:**
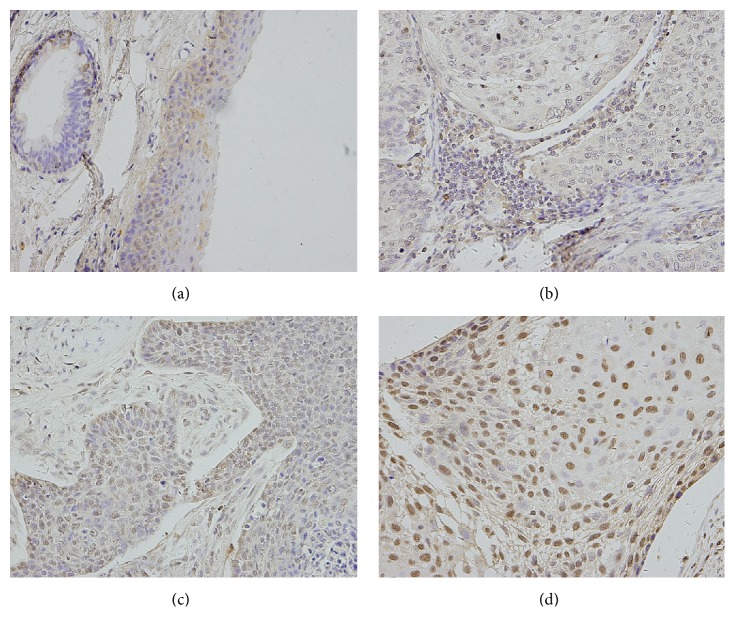
*Expression of TRIM24 protein in head and neck squamous cell carcinoma tissues*. (a) Negative nuclear staining of TRIM24 in normal laryngeal squamous epithelium. (b) Negative TRIM24 expression in a case of well-differentiated HNSCC. (c) Moderate TRIM24 expression in a case of HNSCC. (d) Strong nuclear staining of TRIM24 in poor-differentiated carcinoma. Magnification 400x.

We analyzed the expression of TRIM24 in 100 HNSCC specimens and their normal tissues by immunohistochemistry. Nuclear TRIM24 staining was considered positive staining and we combined staining intensity and percentage to evaluate TRIM24 status. Normal larynx epithelial tissues exhibited negative or weak expression (Figure 1(a)). In contrast, moderate and strong TRIM24 overexpression were observed in 43% (43/100) of HNSCC tissues examined (Figures 1(b)–1(d)).

We analyzed the correlation between TRIM24 overexpression and the clinical factors (Table 1). There was no difference between TRIM24 status and age (*p* = 0.7919), gender (*p* = 0.5324), tumor differentiation (*p* = 0.9867), and nodal metastasis (*p* = 0.1355). The percentages of TRIM24 overexpression in stages I-II and III-IV were 32.8% and 63.6%, respectively. Statistical analysis showed that TRIM24 overexpression correlated with advanced clinical stage of HNSCC (*p* = 0.0034). TRIM24 overexpression also positively correlated with advanced T stage (*p* = 0.0048).

### 3.2. TRIM24 Promotes Proliferation and Invasion of HNSCC Cell Lines

**Figure 2:**
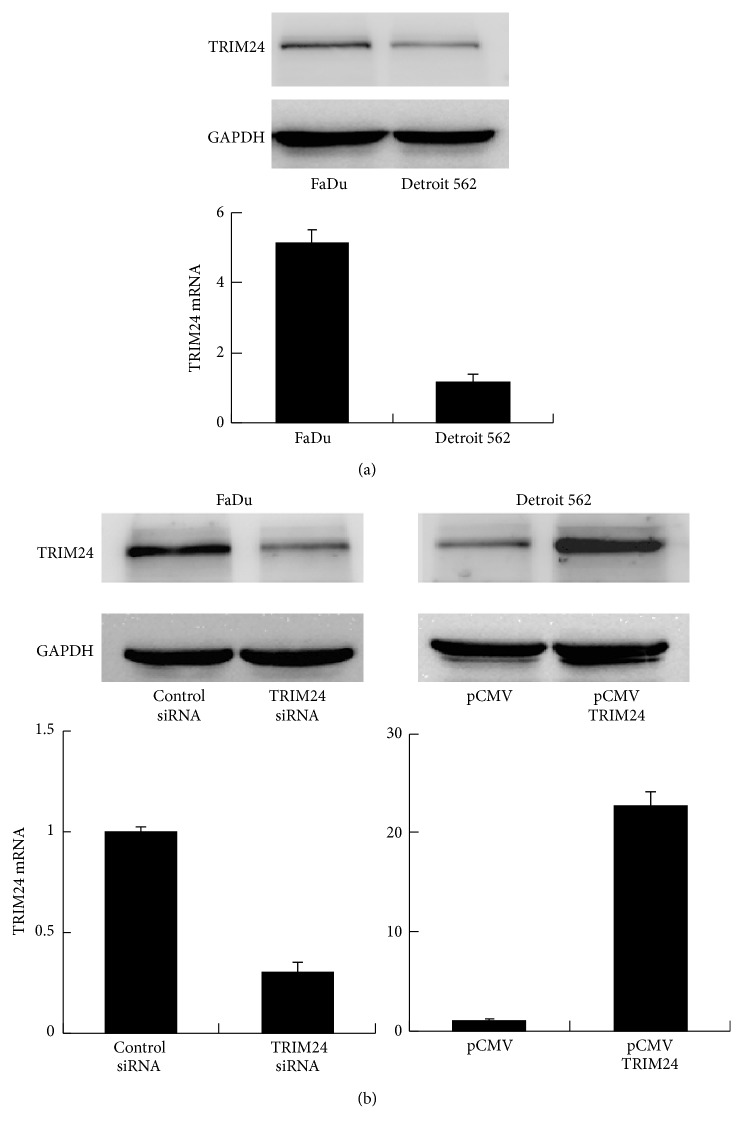
*Efficiency of TRIM24 overexpression and depletion*. (a) Protein and mRNA expression of TRIM24 in HNSCC cell lines KB, FaDu, and Detroit 562. (b) Western blot and real-time RT-PCR analysis showed that TRIM24 plasmid transfection upregulated its mRNA and protein expression, while TRIM24 siRNA downregulated its mRNA and protein levels.

**Figure 3:**
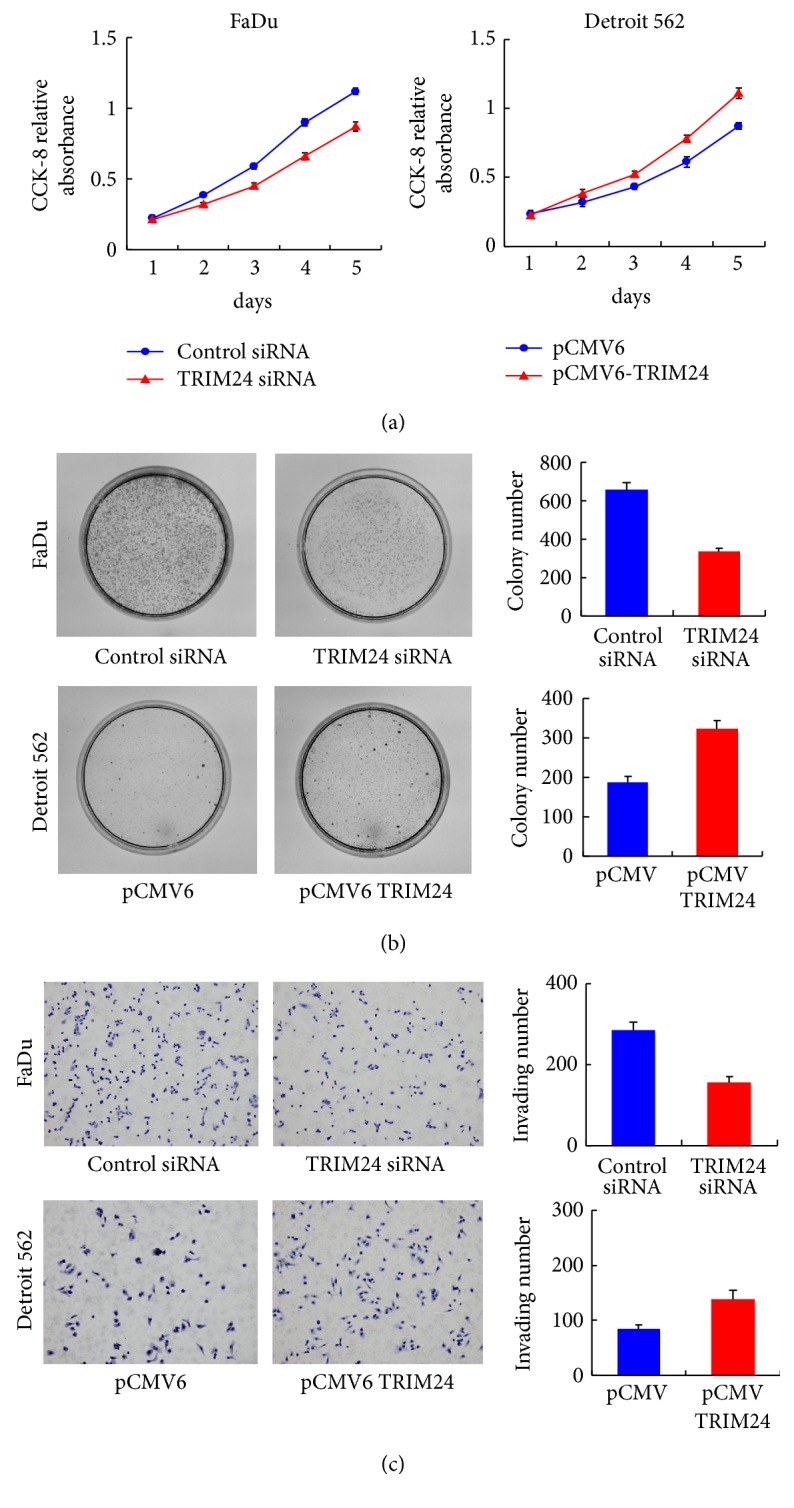
*TRIM24 regulates cell proliferation, colony formation, and invasion*. (a) CCK-8 assay showed that TRIM24 overexpression promoted while its depletion inhibited cell growth rate. (b) Colony formation assay was performed in cells transfected with TRIM24 siRNA and plasmid. An increase of colony number was found in cells transfected with plasmid. A decrease in colony formation was seen in the groups with siRNA treatment in comparison with the controls. (c) TRIM24 overexpression greatly facilitated cell invasion, while TRIM24 depletion remarkably blocked invasion.

We then checked protein and mRNA expression of TRIM24 in HNSCC cell lines FaDu and Detroit 562. As shown in Figure 2(a), FaDu cell line showed relatively high TRIM24 expression while Detroit 562 showed low endogenous TRIM24 expression. In order to investigate the biological functions of TRIM24 in HNSCC cells, we performed gain-of-function and loss-of-function experiments in Detroit 562 and FaDu cell lines. Western blot and real-time RT-PCR analysis confirmed the transfection and knockdown efficiency (Figure 2(b)). We performed CCK-8 and colony formation assays to examine the effect of TRIM24 on proliferation of HNSCC cells. TRIM24 overexpression induced a significant increase of proliferation rate compared with negative control, while its depletion inhibited proliferation rate (Figure 3(a)). The results from colony formation assay demonstrated that TRIM24 transfection significantly upregulated colony number while its depletion downregulated colony number (Figure 2(b)). Together, these results suggested that TRIM24 regulated proliferation of HNSCC cells.

### 3.3. TRIM24 Promotes Invasion and Cell Cycle Progression of HNSCC Cells

**Figure 4:**
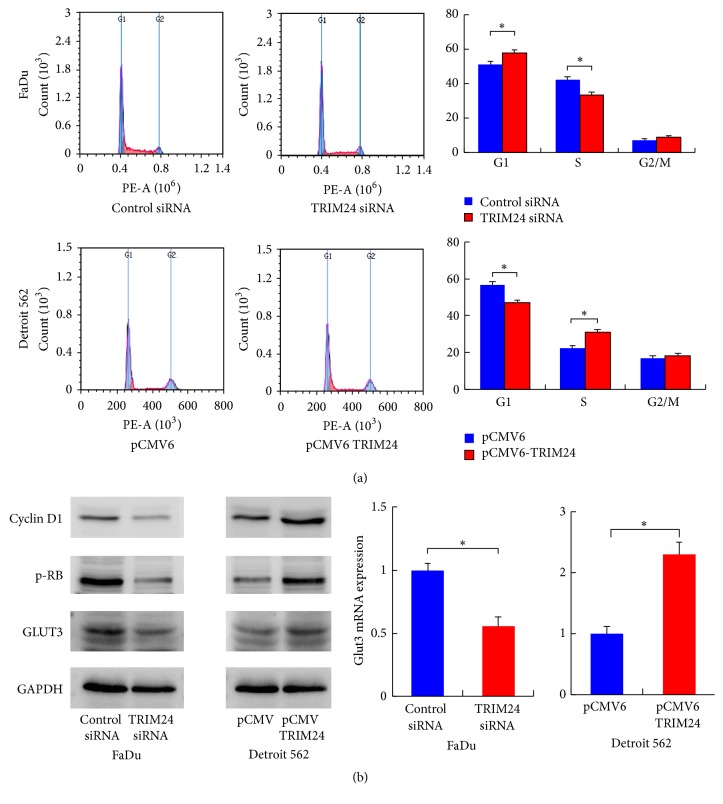
*TRIM24 regulates cell cycle distribution and protein levels of cyclin D1, p-Rb, and GLUT3*. (a) TRIM24 depletion reduced S phase percentage in FaDu cell line while its overexpression upregulated S phase percentage in Detroit 562 cell line. (b) Western blot analysis revealed that knockdown of TRIM24 downregulated while TRIM24 transfection increased the protein levels of cyclin D1, p-Rb, Rb, and GLUT3. TRIM24 downregulated while TRIM24 transfection increased the mRNA level of GLUT3. ^*∗*^*p* < 0.05.

To further evaluate its effect on HNSCC invasion, HNSCC cells transfected with TRIM24 plasmid or TRIM24 specific siRNA were examined by Matrigel invasion assay. Obviously, ectopic TRIM24 expression greatly facilitated cell invasion, while TRIM24 depletion remarkably blocked invasion (Figure 3(c)). These findings indicated that TRIM24 is a potent positive regulator of invasion in head and neck squamous cell carcinoma. In addition, we checked the change of cell cycle distribution after TRIM24 overexpression and depletion. As shown in Figure 4(a), TRIM24 depletion reduced S phase percentage in FaDu cell line while its overexpression upregulated S phase percentage in Detroit 562 cell line (*p* < 0.05). As shown in Figure 4(b), TRIM24 positively regulated cyclin D1 and p-Rb protein expression, which was in accord with cell cycle results. Expression of total Rb was not changed.

### 3.4. TRIM24 Enhances Glucose Metabolism

**Figure 5:**
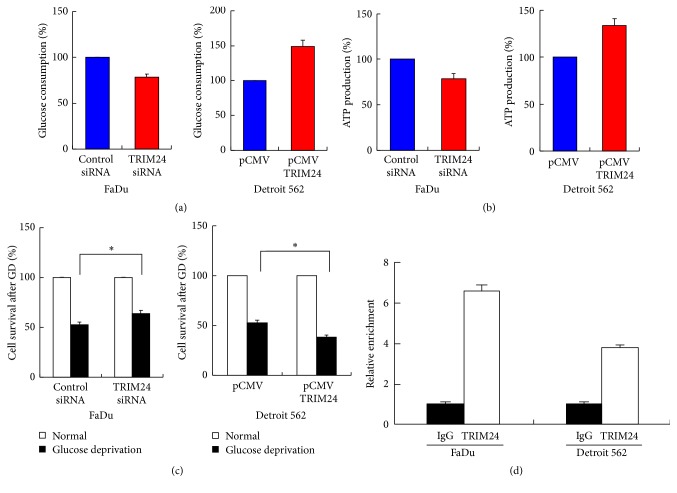
*TRIM24 increases glucose metabolism in HNSCC*. (a) TRIM24 depletion in FaDu cells reduced glucose consumption while TRIM24 overexpression increased glucose consumption in Detroit 562 cells. (b) TRIM24 depletion reduced ATP production in FaDu cells while TRIM24 overexpression upregulated ATP production in Detroit 562 cells. (c) Cell growth was examined after 3 days of glucose deprivation. With glucose deprivation, the cell viability of control cells was higher than that of TRIM24 overexpressed Detroit 562 cells and lower than TRIM24 depleted FaDu cells. (d) ChIP assay was performed in FaDu and Detroit 562 cells. Data were presented as fold enrichment of the TRIM24 antibody signal versus the control IgG, calculated using the comparative Ct method. ^*∗*^Statistical significance.

Glucose uptake is the key step during glucose metabolism. We demonstrated that Detroit 562 cells showed higher glucose consumption compared with control (Figure 5(a)). Accordingly, PCR and western blot demonstrated that TRIM24 induced GLUT3 expression in Detroit 562 cells. These results indicate increased glucose uptake and consumption after TRIM24 overexpression. Aerobic glycolysis of glucose could produce energy in cancer cells. We measured ATP production to indicate the level of aerobic glycolysis. As shown in Figure 5(b), TRIM24 upregulated ATP production while TRIM24 depletion downregulated ATP production. These data indicate that TRIM24 enhances glucose metabolism in HNSCC cells. In addition, we checked mRNA expression of other genes related to glucose metabolism including GLUT1, GLUT2, GLUT4, GLUL, GLS, GOT1, and GOT2. As shown in Supplementary Figure 1A, TRIM24 overexpression did not cause significant mRNA change of these genes. Western blot was performed for GLUT1, GLUT2, and GLUT4, which did not show significant changes (Supplementary Figure 1B).

### 3.5. TRIM24 Increases Sensitivity to Glucose Deprivation and Binds to Cyclin D Promoter

Since TRIM24 upregulation induced a higher level of glucose metabolism, we hypothesized that cells with higher TRIM24 expression might be more dependent on glucose for growth and survival, which means that these cells are more sensitive to glucose deprivation. As shown in Figure 5(c), with glucose deprivation, the cell viability of control cells was higher than that of TRIM24 overexpressed cells, while TRIM24 depletion enhanced cell viability when treated with glucose deprivation.

It is reported that TRIM24 could act as a transcriptional activator [14]. To further explore the mechanisms by which TRIM24 promote the proliferation of HNSCC cells, we investigated if TRIM24 could serve as a transcriptional activator which activates cyclin D. From previous report [14], we noticed ChIP-seq results of TRIM24 in Lncap and K562 cells (Encode project), which revealed that there were potential TRIM24 binding sites in Cyclin D1 promoter, including 5734, 2166, and 64 upstream from the transcription start site. These sites were tested and we found that the -64 position had strong binding activity for TRIM24 (Figure 5(d)).

## 4. Discussion

Recently, a growing number of studies have reported that expression of TRIM24 protein is associated with tumor progression and survival in various cancers. Overexpression of the TRIM24 was found in breast cancer, non-small-cell lung cancer, and malignant glioma. Clinical data demonstrates that TRIM24 correlated with poor patient survival [6, 8, 9, 13, 15]. There is one report showing that TRIM24 overexpression serves as a prognostic factor in HNSCC and that TRIM24 silencing reduced cell proliferation [11]. However, the potential mechanism of TRIM24 in HNSCC cell proliferation remains obscure. Here we demonstrated that TRIM24 was upregulated in 43% of cases of HNSCC and correlated with TNM stage, which is in accord with previous reports, indicating its association with malignant behavior.

To further reveal the roles of TRIM24 in HNSCC cells, plasmid transfection and siRNA transfection were carried out in HNSCC cells. Our results demonstrated that TRIM24 overexpression accelerated while its knockdown inhibited cancer cell proliferation and colony formation. TRIM24 overexpression also facilitated cell cycle progression and upregulated cyclin D1 and p-Rb expression. Cyclin D1 and p-Rb are key regulators of cell cycle progression. These results suggested that TRIM24 promoted HNSCC progression through regulation of cell cycle related proteins. In addition, TRIM24 has been recently reported as a transcription factor in several studies [14, 16]. Accordingly, we showed that TRIM24 could bind to cyclin D1 promoter region using ChIP analysis, which partly elucidate the molecular mechanism for its effect on cell proliferation.

One typical feature of cancer metabolism is switching energy production from oxidative phosphorylation to glycolysis. Elevated glucose uptake, glucose consumption, and lactate production are widely found in various cancers. Upregulation of rate-limiting glucose metabolic enzymes including GLUT family protein may play a central role in elevated glucose consumption and ATP production of cancer cells. Our results demonstrated that TRIM24 overexpression enhanced glucose consumption and ATP production, suggesting enhanced glucose metabolism induced by TRIM24. In addition, after screening several glucose metabolic enzymes (GLUT1/2/3/4, GLUL, GLS, and GOT1/2), we found that TRIM24 was able to induce protein and mRNA of GLUT3, a glucose transporter, which further confirms the fact that TRIM24 regulates glucose metabolism. GLUT3 has been shown to regulate malignant biological behavior of cancer cells. Overexpression of GLUT3 is associated with poor patient survival in lung cancer [17], oral squamous cell carcinoma [18], and laryngeal carcinoma [19]. It is reported that GLUT3 is induced during epithelial-mesenchymal transition and promotes tumor cell proliferation of lung cancer cells [20]. Thus our data suggested that TRIM24 promotes proliferation and glucose metabolism through regulation of GLUT3 in HNSCC cells.

Due to altered glucose metabolism that cancer cells usually have, targeting glucose metabolism to inhibit cancer progression is an attractive approach. Our data indicated that TRIM24 sensitizes HNSCC cells to glucose deprivation, suggesting TRIM24 as a potential therapeutic target for cancer metabolism.

## 5. Conclusions

In summary, we described clinical significance and biological roles of TRIM24 in human HNSCC. Our results show that increased TRIM24 in HNSCC plays an important role in tumor metabolism and progression, possibly through regulation of GLUT3 and cyclin D1. TRIM24 might function as a potential oncoprotein in HNSCC.

## Figures and Tables

**Table 1:** Clinical profile and correlation between the clinicopathological features and TRIM24 expression in HNSCC.

Characteristics	Number of patients	TRIM24 low expression	TRIM24 high expression	*p*
Age				
<60	55	32	23	0.7919
*⩾*60	45	25	20	
Gender				
Male	76	42	34	0.5324
Female	24	15	9	
Differentiation				
Well	59	34	25	0.9867
Moderate	34	19	15	
Poor	7	4	3	
TNM stage				
I + II	67	45	22	0.0034
III + IV	33	12	21	
Lymph node metastasis				
Absent	77	47	30	0.1355
Present	23	10	13	
Tumor stage (T )				
T1 + T2	84	53	31	0.0048
T3 + T4	16	4	12	

## Supplementary Materials

Supplementary Materials Supplementary Figure 1: (A) real-time PCR was performed to examine the change of GLUT1, GLUT2, GLUT4, GLUL, GLS, GOT1, and GOT2. TRIM24 overexpression or knockdown did not cause significant mRNA change of these genes. (B) Western blot of GLUT1, GLUT2, and GLUT4. Change of these proteins was not significant. Supplementary Figure 2: STR testing results obtained from Shanghai cell bank.
